# Effects of probiotic bacterium *Weissella cibaria* CMU on periodontal health and microbiota: a randomised, double-blind, placebo-controlled trial

**DOI:** 10.1186/s12903-020-01231-2

**Published:** 2020-09-02

**Authors:** Mi-Sun Kang, Dong-Suk Lee, Seung-Ah Lee, Myoung-Suk Kim, Seoul-Hee Nam

**Affiliations:** 1 R&D Center, OraPharm Inc., Seoul, 04782 South Korea; 2 School of Nursing, Kangwon National University, Chuncheon, 24341 South Korea; 3 Department of Dental Hygiene, College of Health Sciences, Kangwon National University, 346 Hwangjo-gil, Dogye-up, Samcheok-si, Gangwon-do 25949 South Korea

**Keywords:** Probiotics, *Weissella cibaria*, Periodontal health, Bleeding, Microbiota, Clinical study

## Abstract

**Background:**

*Weissella cibaria* CMU (oraCMU) has been commercially available in the market for several years as oral care probiotics. The present study aimed to evaluate the effects of oraCMU-containing tablets on periodontal health and oral microbiota.

**Methods:**

A randomised, double-blind, placebo-controlled trial was conducted in 92 adults without periodontitis (20–39 years of age). All subjects received dental scaling and root planing, and were randomly assigned to either probiotic or placebo groups. The tablets were administered once daily for 8 weeks. Periodontal clinical parameters included bleeding on probing (BOP), probing depth (PD), gingival index (GI), and plaque index (PI). In addition, microbiota in the gingival sulcus were analysed.

**Results:**

BOP improved more in the probiotic group over 8 weeks. There were statistically significant differences in BOP of the maxilla buccal and lingual sites between the groups during the intervention (*P* < 0.05). No significant inter-group differences in PD, GI, and PI were observed during the intervention. Oral bacteria were observed to be fewer in the probiotic group. There was a significant change in levels of *Fusobacterium nucleatum* at four and 8 weeks between the two groups. Besides, there were significant differences at 8 weeks in levels of *Staphylococcus aureus*.

**Conclusions:**

We reported an improvement in BOP and microbial environment and demonstrated the antimicrobial activity of oraCMU against *F. nucleatum*. Thus, its supplementation may contribute to overall oral health.

**Trial registration:**

Ethical issues approved by the Kangwon National University Institutional Review Board with a number of KWNUIRB-2018-05-003-005 and CRIS code Number of KCT0005078 were retrospectively registered on 06/02/2020. This study was conducted in the period of July to November 2018.

## Background

Periodontitis is a widespread inflammatory disease that affects the structure and supporting tissues of the teeth and causes the destruction of the connective tissue [1]. Periodontal disease is also a plaque-related infectious disease caused by pathogenic biofilm accumulating on the dental surface and oral mucosa. It is considered one of the most common chronic diseases worldwide, is caused by plaque-related bacteria, and is a major cause of tooth loss [2]. It is regarded as a risk factor for various systemic diseases such as diabetes, rheumatoid arthritis, and osteoporosis [3].

Changes in bacterial distribution occur when normal gingival sulcus transforms into pathological periodontal pockets. These bacteria have various pathogenic properties that colonise the gingival space, evade the defence system of the host, and damage tissues [4]. Recently, the biological and physiological functions of probiotic bacteria in relation to dentistry have been revealed [5]. Probiotic bacteria act through various mechanisms, such as competitive inhibition of attachment and growth of pathogens, lowering metabolism of environmental pH, direct antimicrobial effect through the production of antimicrobial substances, and modulation of local and systemic immune responses [6]. Thus, probiotics might be advantageous in preventing or treating oral diseases such as caries, gingivitis, or periodontitis by improving the environment of oral microbiota in the oral cavity.

Probiotics are defined as “live microorganisms which, when administered in adequate amounts, confer a health benefit on the host” [7]. Probiotic bacteria mainly include lactic acid bacteria (LAB), such as the genus *Lactobacillus* and *Bifidobacterium* [8]. Koll-Klais et al. [9] found that the genus *Lactobacillus*, which resides in the oral cavity, plays an important role in the ecological balance of the oral cavity. Several strains of *Weissella cibaria* have also shown probiotic potential [10, 11]. The genus *Weissella* is a Gram-positive LAB and formerly considered a species of the *Leuconostoc paramesenteroides* group [12]. In particular, *W. cibaria* was first classified in a taxonomic study in 2002 and has been denoted as a dominant species in fermented foods such as kimchi [13].

*W. cibaria* strains CMU, CMS1, CMS2, and CMS3 have shown probiotic potential in the field of dentistry, owing to their inhibitory effect on *Streptococcus mutans* biofilm formation and volatile sulfur compound (VSC) formation [14–16]. These strains have been isolated from the saliva of children ages 4 to 7 years old with good oral health [14]. *W. cibaria* CMU has been reported to inhibit the production of interleukin (IL)-6 and IL-8, which are pro-inflammatory cytokines induced by periodontal bacteria such as *Fusobacterium nucleatum* in oral epithelial cells [16]. Hydrogen peroxide and organic acids (e.g., lactic acid, acetic acid, and citric acid) from *W. cibaria* CMU have been known to be involved in antimicrobial activity [17].

This study aimed to identify the oral health improvement effects on gum health and oral microbial changes with the use of *W. cibaria* CMU tablets. The research question was “Can oral sucking intake of *W. cibaria* CMU improve oral health indexes (BOP, PD, PI, GI) and decrease oral pathogen as time passed, compared to control group?”

## Methods

### Ethical consideration

This study was conducted in accordance with the International Council for Harmonization of Technical Requirements for Pharmaceuticals for Human Use (ICH) guidelines. Approval for the study was obtained from the Kangwon National University (KNU) Institutional Review Board (KWNUIRB-2018-05-003-005, Chuncheon, Korea). The purpose and procedure of the study were explained to all participants. Participants were also informed that refusal to participate would not disadvantage them in any way, and they were free to withdraw from the study at any time. Written informed consent was obtained from all participants prior to enrolment.

### Participants

Participants were recruited through an offline poster and an online public notice using social network services aimed at undergraduate students, graduate students, and school personnel at KNU (Chuncheon, Korea). The sample size was calculated using the G * Power 3.1 programme. The number of participants needed for the independent t-test with significance level α = 0.05 bilateral test, power = 0.8, and effect size = 0.7 was 68. The initial sample size was planned at 96, considering a dropout rate of 40%; 100 participants were enrolled in the current study. The effect size was set to medium or high based on previous studies that reported the effects of *W. cibaria* CMU administration. The dropout rate was set at high as the subjects were college students or working adults. Random allocation sequence for test group or placebo group was generated via Microsoft Excel [18] by a research assistant not participated in this study intervention. The formula was following: If (Rank (B2, $B$2: $B$101) > 50, 1, 0). Sequential numbered opaque sealed envelopes was used until assignment and opened sequentially at screening. Other 3 research assistants enrolled and assigned participants to intervention and there was not any restriction in random allocation. A total of 100 subjects were screened, and 92 were randomly assigned to the probiotic test group (*n* = 49) or placebo control group (*n* = 43), after excluding eight subjects who did not meet the inclusion criteria or refused to participate during the two-week run-in period. Twenty-four additional subjects were excluded from the eight-week intervention phase, and data were finally analysed for 68 subjects. The inclusion criteria were as follows: subjects who were able to comply with the protocol, over 20 years of age with more than 20 natural teeth, no tongue problems, no gum diseases, and with oral VSC concentration of 1.5 ng/10 mL or more. The exclusion criteria were as follows: subjects who had received antibiotic treatment within the previous month; those currently visiting their dentists for treatment; with adverse reactions to lactose or fermented milk products; consistently using probiotic supplements; with a dry mouth; with systemic diseases that would cause halitosis; who could not see or hear sufficiently; with mental illness; and those who had participated in another clinical trial within the previous month.

### Study treatments

The 800 mg probiotic tablet contained 1.0 × 10^8^ colony forming units (CFU)/g of *W. cibaria* CMU (oraCMU) provided by OraPharm Inc. (Seoul, Korea). Other ingredients included isomalt, sucralose, peppermint flavour, maltodextrin, and magnesium stearate. The placebo was a tablet from the same manufacturer with the same taste, texture, and appearance lacking oraCMU. Subjects were instructed to melt and suck one tablet in their mouth every night before bedtime after brushing their teeth. Water or food was prohibited after the treatment. The study treatment was conducted for 8 weeks.

### Study design and protocol

A randomised double-blind, placebo-controlled trial was performed. To secure the homogeneity of the oral condition of the subjects, they were required to visit M Dental Clinic in Chuncheon 1 week prior to starting the test treatment, received an oral examination by a dentist, and underwent dental scaling and root planing (SRP). After SRP, they had a recovery period of 1 week for the regeneration of the gums. One week after the recovery period, the probiotic tablet was administered to the experimental group, and a placebo tablet of the same shape was administered to the control group. The typical maxillary and mandibular teeth, including the maxillary right first molar (#16), maxillary left central incisor (#21), maxillary left first premolar (#24), mandibular left first molar (# 36), mandibular right central incisor (#41), and mandibular right first premolar (#44) were selected for clinical examination. Clinical examination including probing depth (PD), bleeding on probing (BOP), plaque index (PI), and gingival index (GI) was performed at three visits (baseline, at four, and 8 weeks). The measurement of bacteria in subgingival plaque was also performed in the three visits.

During the experimental period, all subjects were provided with the same type of toothpaste and toothbrush and were instructed to use them throughout the study. All the interventions were performed in double-blind state: participants were distinguished by only registration number, intervention providers (research assistants) neither know who was test group or placebo group as well. The study was conducted in the period of July to November 2018.

### PD

PD was used to evaluate the probing pocket depth by measuring the distance from the marginal gingiva to the epithelial attachment region using a probe, depending on the observed changes of the gingival sulcus [19].

### Bop

The criteria used for GI evaluation were applied to teeth and tooth surfaces to assess BOP. The presence of bleeding from the base of the gingival sulcus was denoted as (+), and its absence was denoted as (−). The incidence of bleeding (%BOP) was calculated [20].

### Pi

Using Loe and Silness PI technique [21], each tooth surface was coloured with a red colourant, and the tooth surface was divided into two parts (occlusal and gingival surfaces) to measure plaque accumulation and thickness of the gingival margin. The evaluation criteria were: 0 = no plaque; 1 = thinly attached to the gingival margin and apparent after lightly scraping with a probe or applying a tooth colourant; 2 = moderate plaque that can be visually recognised along the gingival margin; and 3 = thick plaque accumulation in the gingival pockets, as well as gingival margin and tooth surface. The total PI score per subject was calculated with the average value of each tooth surface.

### GI

GI was evaluated at the proximal, distal, buccal, and lingual sites and each site was assigned 0–3 points [22]: 0 = healthy gingiva; 1 = gingivitis with a slight colour change and slight swelling, but without bleeding by mild irritation; 2 = gingivitis with redness, swelling, and bleeding by mild irritation; and 3 = advanced inflammation with marked redness and swelling and the possibility of ulceration and natural bleeding. The total PI score per subject was calculated with the average value of each tooth surface.

### Microbiological analysis

#15 paper points were inserted to the gingival sulcus of four sites of two maxillary teeth (anterior and posterior) and two mandibular teeth (anterior and posterior) of subjects with PD less than 4 mm for 10 s and were then placed in a 1.5 mL tube. They were immediately stored at − 20 °C until just before analysis. DNA was extracted from the collected paper points using the AccuPrep Universal RNA Extraction Kit (Bioneer, Daejeon, Korea). The extraction was performed according to the manufacturer’s instructions. OligoMix (YD Global Life Science Co., Ltd., Seongnam, Korea) and three oligonucleotides (forward primer, reverse primer, and probe) (Table 1) that react specifically to each bacterium were used [23]. To prepare the polymerase chain reaction (PCR) reaction sample, 9 μL of OligoMix, 10 μL of 2x probe qPCR mix (Takara Bio Inc., Shiga, Japan), and 1 μL of template DNA were combined. A 96-well plate with the PCR reaction sample was placed in the CFX96 Touch Real-Time PCR Detection System (Bio-Rad, Hercules, USA) to amplify the DNA. The conditions of PCR were as follows: initial denaturation at 95 °C for 30 s, denaturation at 95 °C for 10 s, and annealing for 30 s at 62 °C with 40 repeated cycles. The cycle threshold (Ct) value was calculated using the Bio-Rad CFX Manager Software program, and the number of copies was derived by plotting the Ct value in the standard curve of each bacterium.

**Table 1:** Primers and probes used in the real-time PCR assays

Bacteria	Target genes	Primers/Probe sets	Amplicon size (bp)
*Aggregatibacter actinomycetemcomitans*	leukotoxin gene	5′-CGGGGCTTTCTACTACGGGA-3′	123
		5′-ATGCCTCAAGCATTCTCGCA-3′	
		5′-FAM-GGTCAGCTTGGCAATCAGCCC-BHQ1–3’	
*Campylobacter rectus*	*groEL* gene	5′-AAATTTAAGCGGCGACGAGG-3’	132
		5′-TCCTTGCTCACGCTTACGGA-3’	
		5′-HEX-GGCTTTGACGCGGGCGTAGT-BHQ1–3’	
*Eikenella corrodens*	proline iminopeptidase (*pip*) gene	5′-GCCAACTGCTGCTGGAAGTG-3’	110
		5′-GCCGCTGATTTCGGAGAGTT-3’	
		5′-HEX- ACAGCCATCGGCACAGGCAT-BHQ1–3’	
*Fusobacterium nucleatum*	16S ribosomal RNA gene	5′-GGCTGTCGTCAGCTCGTGTC-3’	114
		5′-CTCATCGCAGGCAGTATCGC-3’	
		5′-FAM-AACGAGCGCAACCCCTTTCG-BHQ1–3’	
*Porphyromonas gingivalis*	hemagglutinin (*phg*) gene	5′-ACACGGTGTATCGTGACGGC-3’	119
		5′-GCCGGCTGCGTACTTAACCT-3’	
		5′-HEX-CGACCTACCGCGATGCAGGA-BHQ1–3’	
*Prevotella intermedia*	hemagglutinin (*phg*) gene	5′-CACACGCTGGCGAAACCTAC-3’	143
		5′-CACGTGGCGTTGCTTCTTTC-3’	
		5′-HEX-CCGAAGATGCGCCGTTGAAC-BHQ1–3’	
*Prevotella nigrescens*	gyrase subunit B (*gyrB*) gene	5′-AGCAAGCTGTAGGCGAGGCT-3’	132
		5′-GCTGAACACTTTCGCGTGCT-3’	
		5′-Texas Red-GCTCGTATTGCAGCCCGCAA-BHQ2–3’	
*Tannerella forsythia*	karilysin protease gene	5′-TGGCAAATCGCTCATCATCC-3’	140
		5′-TTCCATGTTCCCCAACCACA-3’	
		5′-Texas Red-CCATTAAGCCCATTGCCCGG-BHQ2–3’	
*Treponema denticola*	oligopeptidase B (*opdB*) gene	5′-AGAAAGGCTTTGGGCGACAG-3’	127
		5′-GCTGGAGCCGTAGCTTCCAT-3’	
		5′-Cy5-CGGGTCCTCACCCGCTCTTC-BHQ2–3’	
*Actinomyces viscosus*	sialidase (*nanH*) gene	5′-GCTCCCTCATGCTCAACTCG-3’	140
		5′-GATGATCTGGGCGTTGTCCA-3’	
		5′-Texas Red-GAGCCGGTCCCCGACAAGAA-BHQ2–3’	
*Enterococcus faecalis*	gelatinase (*gelE*)-serine protease (*sprE*) operon	5′-GATGGCAGTGCGACACCATT-3’	101
		5′-CGATCGTTTTGTTTGCCGAC-3’	
		5′-HEX-GCTTTTTCCCCGAATGCGGA-BHQ1–3’	
*Eubacterium nodatum*	hypothetical protein	5′-TGCTTGCCGGTGACTTAGGA-3’	130
		5′-AAACCGGGCTCAACAACCAT-3’	
		5′-Texas Red-TTGAGGAGCCGGTGACTTTGG-BHQ2–3’	
Group of *Streptococcus*	16S ribosomal RNA gene	5′-GTACAACGAGTCGCAAGCCG-3’	149
		5′-TACAAGGCCCGGGAACGTAT-3’	
		5′-FAM-TAATCGCGGATCAGCACGCC-BHQ1–3’	
*Parvimonas micra*	16S ribosomal RNA gene	5′-GAGGAATACCGGTGGCGAAG-3’	148
		5′-GGCACCGAGATTTGACTCCC-3’	
		5′-FAM-GGTACGAAAGCGTGGGGAGCA-BHQ1–3’	
*Staphylococcus aureus*	clumping factor A (*clfA*) gene	5′-GCGCAAGTAACGAAAGCAAAA-3’	132
		5′-GATTTTGCGCCACACTCGTT-3’	
		5′-FAM-TGCTGCACCTAAAACAGACGACACA-BHQ1–3’	
*Streptococcus mutans*	mannitol-specific enzyme II (*mtlA*) gene	5′-CAGCGCATTCAACACAAGCA-3’	103
		5′-TGTCCCATCGTTGCTGAACC-3’	
		5′-HEX-TGCGGTCGTTTTTGCTCATGG-BHQ1–3’	

### Statistical analysis

All study results were evaluated according to the per protocol analysis. All data were analysed using SPSS 21.0 for Windows (IBM Corp., Armonk, USA). An independent *t*-test, *x*^2^ test, and Fisher’s exact test were used for confirmation of homogeneity between the two groups at baseline. The Kolmogorov-Smirnov test and the Shapiro-Wilk test were used to check the normality of the data. For the clinical variables and the concentrations of bacteria, an independent *t*-test allowed a comparison between probiotic and placebo groups. Otherwise, the Mann-Whitney *U* test was used. A value of *P* ≤ 0.05 was considered statistically significant.

## Results

### Study population

**Fig. 1:**
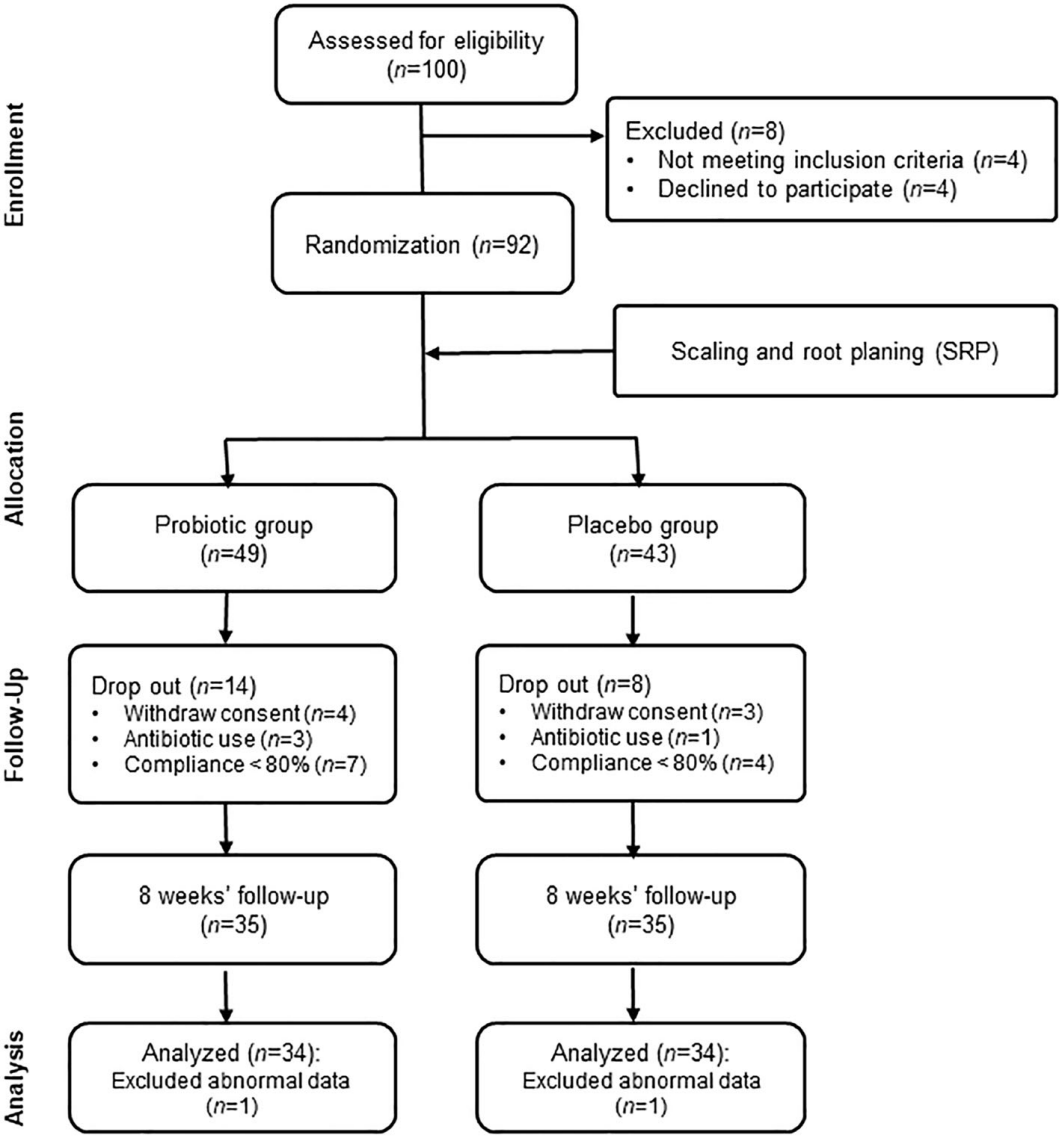
CONSORT flow diagram

CONSORT flow diagram of this study is shown in Fig. 1. The baseline characteristics of the study subjects are shown in Table 2. No significant differences were observed between the groups (*P* > 0.05).

**Table 2:** Baseline characteristics of the subject in the probiotic and placebo groups *N* = 68

Characteristics		Probiotic (*n* = 34) N (%)	Placebo (*n* = 34) N (%)	x^2^ or Z or t	*P*-value
Age (year) (mean ± SD)		23.4 ± 2.9	23.6 ± 3.4	− 0.230^a^	.817
Gender	Male	24 (70.6)	19 (55.9)	1.580	.209
	Female	10 (29.4)	15 (44.1)		
Drinking		24 (70.6)	23 (67.6)	0.066	.793
Smoking		3 (8.8)	6 (17.6)	1.150^a^	.476
Brushing/day	None	1 (2.9)	0 (0.0)	3.190^a^	.530
	Once	1 (2.9)	3 (8.8)		
	Two times	13 (38.2)	10 (29.4)		
	Three times	10 (29.4)	14 (41.2)		
	Four or more	9 (26.5)	7 (20.6)		

^a^Fisher’s exact test

### Clinical outcomes

**Fig. 2:**
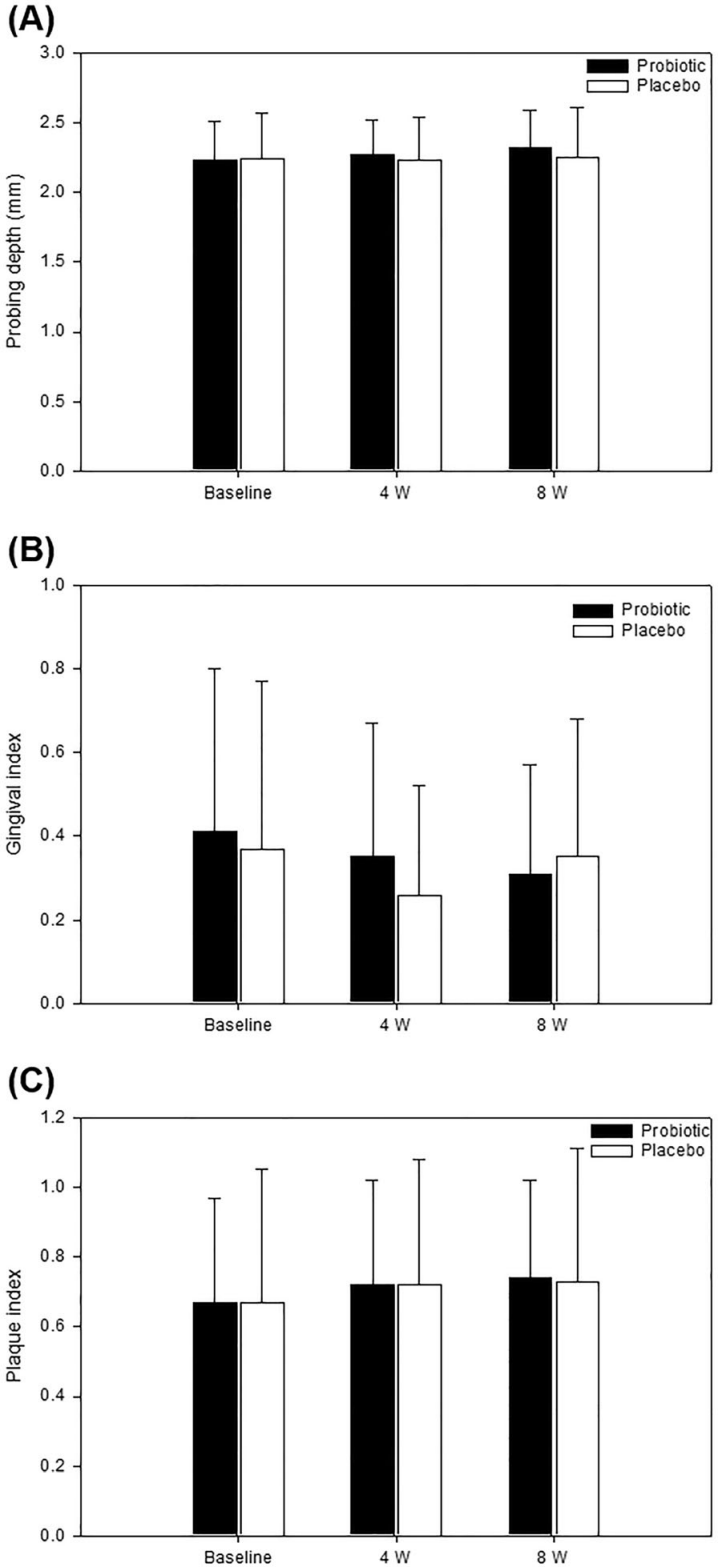
Mean probing depth (**a**), gingival index (**b**), and plaque index (**c**) outcome measures at baseline, 4, and 8 weeks. No statistically significant difference was observed between the groups

As shown in Table 3, both groups had similar mean BOP at baseline. Treatment resulted in a significant reduction in BOP from baseline (*P* < 0.05). There was a statistically significant difference in the BOP mean values between groups in the maxillary buccal site at week 8 and in the maxillary lingual site at week 4 (*P* < 0.05). However, there were no statistically significant inter-group differences in BOP reduction as well as the mean BOP in all the observed teeth. As shown in Fig. 2, no significant inter-group differences were observed at baseline, 4 weeks, and 8 weeks for PD, GI, and PI (*P* > 0.05).

**Table 3:** Percentages of sites with bleeding on probing (BOP) measurements at baseline, 4, and 8 weeks *N* = 68

Teeth site		Timepoint	Mean ± SD			Delta mean ± SD		
			Probiotic (*n* = 34)	Placebo (*n* = 34)	*P* -value^#^	Probiotic (*n* = 34)	Placebo (*n* = 34)	*P*-value^#^
Overall		Baseline	16.21 ± 14.23	19.15 ± 17.88	NS			
		4 weeks	7.56 ± 13.20^a^	11.47 ± 14.06^a^	NS	−8.59 ± 14.78	−7.56 ± 13.97	NS
		8 weeks	6.29 ± 8.54^a^	9.74 ± 14.26^a^	NS	−9.82 ± 14.36	−9.35 ± 13.58	NS
Maxilla	Buccal	Baseline	13.68 ± 24.77	14.68 ± 23.52	NS			
		4 weeks	5.85 ± 19.15	8.74 ± 14.78	NS	−7.82 ± 27.33	−5.88 ± 23.80	NS
		8 weeks	1.97 ± 11.49^a^	7.79 ± 16.48	**.030**	−11.74 ± 25.77	−6.85 ± 22.82	NS
	Lingual	Baseline	19.53 ± 28.55	26.47 ± 30.55	NS			
		4 weeks	6.85 ± 17.96^a^	15.62 ± 22.06^a^	**.038**	−12.71 ± 27.18	−10.71 ± 26.81	NS
		8 weeks	6.82 ± 15.91^a^	7.79 ± 20.14^a^	NS	− 12.71 ± 25.90	−18.56 ± 28.61	NS
Mandibular	Buccal	Baseline	8.74 ± 14.78	10.71 ± 17.75	NS			
		4 weeks	4.85 ± 11.86	6.85 ± 21.37	NS	−3.88 ± 17.73	− 3.88 ± 19.51	NS
		8 weeks	5.82 ± 12.77	8.79 ± 18.90	NS	−2.91 ± 20.50	−1.94 ± 16.13	NS
	Lingual	Baseline	22.41 ± 24.20	24.44 ± 25.09	NS			
		4 weeks	12.74 ± 23.28	14.65 ± 22.00	NS	−9.74 ± 31.18	−9.79 ± 27.77	NS
		8 weeks	10.68 ± 15.67^a^	14.65 ± 24.86	NS	−11.68 ± 26.90	− 9.79 ± 28.94	NS

Values are mean ± standard deviation

^a^Significant differences from baseline

^#^Significant differences between the groups: *P* < .05, significant (bold); not significant (NS)

### Microbiological outcomes in subgingival plaque

The Gram-negative and -positive oral bacterial data in subgingival plaques are presented in Tables 4 and 5, respectively. As shown in Table 4, similar findings on Gram-negative oral bacteria were observed in both groups at baseline (*P* > 0.05). After four and 8 weeks, significant differences in the number of *F. nucleatum* were observed in the groups (*P* < 0.05). Most Gram-negative oral bacteria decreased in the probiotic group at four and 8 weeks. Among the Gram-positive oral bacteria, *Actinomyces viscosus,* and a group of *Streptococcus* (GS) were higher in the probiotic group than those in the control group (*P* < 0.05). However, GS significantly decreased between baseline and 4 weeks in the probiotic group (*P* < 0.05), while there was a significant increase in *A. viscosus* in the placebo group (*P* < 0.05). Both bacteria showed statistically significant differences among the groups over 4 weeks (*P* < 0.05). The number of *Staphylococcus aureus* in the placebo group significantly increased compared to the baseline at week 8, and this was significantly higher than that of the probiotic group (*P* < 0.05; Table 5).

**Table 4:** Gram-negative bacterial measurements in subgingival plaque at baseline, 4, and 8 weeks *N* = 68

Variables	Timepoint	Mean (Log_10_ DNA copy N) ± SD			Delta mean (Log_10_ DNA copy N) ± SD		
		Probiotic (*n* = 34)	Placebo (*n* = 34)	*P*-value^#^	Probiotic (*n* = 34)	Placebo (*n* = 34)	*P*-value^#^
*Aggregatibacter actinomycetemcomitans*	Baseline	0.07 ± 0.42	0.00 ± 0.00	NS			
	4 weeks	0.15 ± 0.90	0.00 ± 0.00	NS	0.08 ± 0.48	0.00 ± 0.00	NS
	8 weeks	0.13 ± 0.76	0.06 ± 0.35	NS	0.06 ± 0.35	0.06 ± 0.35	NS
*Campylobacter rectus*	Baseline	0.54 ± 1.06	0.68 ± 1.18	NS			
	4 weeks	0.91 ± 1.27	1.31 ± 1.70^a^	NS	0.37 ± 1.40	0.62 ± 1.27	NS
	8 weeks	1.59 ± 1.67^a^	2.03 ± 2.08^a^	NS	1.05 ± 1.68	1.35 ± 1.58	NS
*Eikenella corrodens*	Baseline	0.32 ± 0.68	0.30 ± 0.89	NS			
	4 weeks	0.27 ± 0.61	0.38 ± 0.79	NS	−0.05 ± 0.97	0.08 ± 0.70	NS
	8 weeks	0.50 ± 0.96	0.86 ± 1.17^a^	NS	0.18 ± 0.93	0.57 ± 1.03	NS
*Fusobacterium nucleatum*	Baseline	4.49 ± 1.09	4.05 ± 1.05	NS			
	4 weeks	4.41 ± 1.09	4.64 ± 1.05^a^	NS	−0.08 ± 1.20	0.59 ± 1.10	**.035**
	8 weeks	5.12 ± 0.84^a^	5.25 ± 1.07^a^	NS	0.63 ± 1.16	1.20 ± 0.91	**.030**
*Porphyromonas gingivalis*	Baseline	0.17 ± 0.82	0.26 ± 0.72	NS			
	4 weeks	0.14 ± 0.73	0.48 ± 1.29	NS	−0.03 ± 1.04	0.21 ± 1.03	NS
	8 weeks	0.15 ± 0.62	0.69 ± 1.82	NS	− 0.02 ± 0.46	0.43 ± 1.32	NS
*Prevotella intermedia*	Baseline	0.62 ± 1.27	0.67 ± 1.24	NS			
	4 weeks	0.75 ± 1.22	0.77 ± 1.56	NS	0.13 ± 1.14	0.10 ± 0.86	NS
	8 weeks	1.23 ± 1.58^a^	1.20 ± 1.93^a^	NS	0.61 ± 1.32	0.54 ± 1.05	NS
*Prevotella nigrescens*	Baseline	1.73 ± 1.48	1.28 ± 1.22	NS			
	4 weeks	2.04 ± 1.51	1.91 ± 1.53^a^	NS	0.32 ± 1.75	0.63 ± 1.50	NS
	8 weeks	2.82 ± 1.55^a^	2.72 ± 1.56^a^	NS	1.09 ± 1.61	1.44 ± 1.52	NS
*Tannerella forsythia*	Baseline	0.53 ± 1.22	0.90 ± 1.38	NS			
	4 weeks	0.66 ± 1.17	1.36 ± 1.90	NS	0.13 ± 1.43	0.46 ± 1.43	NS
	8 weeks	0.99 ± 1.79	1.83 ± 2.29^a^	NS	0.46 ± 1.60	0.93 ± 1.59	NS
*Treponema denticola*	Baseline	0.50 ± 1.09	0.67 ± 1.34	NS			
	4 weeks	1.01 ± 1.59	1.06 ± 1.79	NS	0.51 ± 1.71	0.39 ± 1.37	NS
	8 weeks	1.77 ± 1.87^a^	1.49 ± 2.11^a^	NS	1.27 ± 1.55	0.82 ± 1.43	NS

Values are mean ± standard deviation

^a^Significant differences from baseline

^#^Significant differences between the groups: *P* < 0.05, significant (bold); not significant (NS)

**Table 5:** Gram-positive bacterial measurements in subgingival plaque at baseline, 4, and 8 weeks *N* = 68

Variables	Timepoint	Mean (Log_10_ DNA copy N) ± SD			Delta mean (Log_10_ DNA copy N) ± SD		
		Probiotic (*n* = 34)	Placebo (*n* = 34)	*P* -value^#^	Probiotic (*n* = 34)	Placebo (*n* = 34)	*P* -value^#^
*Actinomyces viscosus*	Baseline	5.42 ± 0.58	4.99 ± 0.64	**.003**			
	4 weeks	5.22 ± 0.48	5.36 ± 0.42^a^	NS	−0.20 ± 0.62	0.37 ± 0.64	**.001**
	8 weeks	5.69 ± 0.48	5.51 ± 0.46^a^	NS	0.27 ± 0.74	0.52 ± 0.54	NS
*Enterococcus faecalis*	Baseline	0.12 ± 0.48	0.07 ± 0.33	NS			
	4 weeks	0.09 ± 0.38	0.20 ± 0.55	NS	−0.03 ± 0.63	0.13 ± 0.50	NS
	8 weeks	0.21 ± 0.60	0.19 ± 0.75	NS	0.09 ± 0.77	0.12 ± 0.83	NS
*Eubacterium nodatum*	Baseline	0.26 ± 0.68	0.39 ± 1.07	NS			
	4 weeks	0.21 ± 0.55	0.43 ± 1.09	NS	−0.05 ± 0.89	0.04 ± 1.07	NS
	8 weeks	0.65 ± 1.46	0.72 ± 1.42^a^	NS	0.39 ± 1.53	0.33 ± 1.30	NS
Group of *Streptococcus*	Baseline	4.95 ± 0.75	4.58 ± 0.74	**.025**			
	4 weeks	4.39 ± 0.56^a^	4.50 ± 0.52	NS	−0.57 ± 0.73	−0.08 ± 0.71	**.008**
	8 weeks	4.95 ± 0.72	4.75 ± 0.65	NS	− 0.01 ± 0.98	0.17 ± 0.60	NS
*Parvimonas micra*	Baseline	1.58 ± 1.19	1.26 ± 1.18	NS			
	4 weeks	1.52 ± 1.32	1.61 ± 1.40	NS	−0.06 ± 1.72	0.36 ± 1.21	NS
	8 weeks	2.35 ± 1.41^a^	2.30 ± 1.46^a^	NS	0.77 ± 1.63	1.05 ± 0.91	NS
*Staphylococcus aureus*	Baseline	0.69 ± 0.91	0.41 ± 0.84	NS			
	4 weeks	0.61 ± 0.95	0.53 ± 0.90	NS	−0.08 ± 1.35	0.12 ± 1.31	NS
	8 weeks	0.40 ± 0.82	0.97 ± 1.12^a^	**.023**	− 0.29 ± 1.26	0.56 ± 1.23	**.014**
*Streptococcus mutans*	Baseline	0.41 ± 0.80	0.11 ± 0.39	NS			
	4 weeks	0.37 ± 0.88	0.29 ± 0.69	NS	−0.04 ± 0.94	0.18 ± 0.82	NS
	8 weeks	0.54 ± 0.96	0.46 ± 0.90	NS	0.13 ± 0.97	0.36 ± 1.03	NS

Values are mean ± standard deviation

^a^Significant differences from baseline

^#^Significant differences between the groups: *P* < .05, significant (bold); not significant (NS)

## Discussion

There is an increased interest in both oral and systemic health as oral health is an important factor that affects the quality of life. Therefore, preventive care is recognised as important in oral health. Since the demand for the prevention of oral disease and the promotion of oral health is increasing from a therapeutic viewpoint of only treating tooth discomfort, patients are likely more inclined to receive a higher quality oral health promotion service.

Research on probiotics as an alternative therapy for oral care has been actively conducted [5]. Probiotics refer to live bacteria that are beneficial to the host when ingested at an appropriate amount [7]. They have been known for their intestinal effects [6], but recently, their function has been known to be extended to strengthening the immune system, reducing cholesterol level, enhancing vaginal health, and improving skin health [24, 25].

In the field of dentistry, probiotics have been recognised to be crucial for the prevention of caries by reducing *S. mutans* [26]. In addition, the ingestion of probiotics can reduce the level of *Candida* in the oral cavity, thus controlling oral yeast infection. As adjuvant therapy for gingivitis or peri-implantitis, *Lactobacillus reuteri* and *L. brevis* have been studied as oral care probiotics [27].

*W. cibaria* CMU is a Gram-positive, non-spore-forming, non-motile, heterofermentative, catalase-negative, rod-shaped lactic acid bacterium [15]. This strain is known to inhibit caries by converting the insoluble glucan of *S. mutans*, a caries-inducing bacterium, into water-soluble dextran [14]. Furthermore, this strain has been reported to exhibit in vitro antimicrobial activity against *Aggregatibacter actinomycetemcomitans, F. nucleatum, Porphyromonas gingivalis, Tannerella forsythia,* and *Treponema denticola* [17]. In addition, the *W. cibaria* strains have been reported to be advantageous due to their competitive attachment to oral epithelial cells with periodontal pathogens in the oral cavity [16]. In particular, they can function as oral care probiotics as they do not produce strong acids, thereby resulting in a low risk of dental caries among the LAB. The oral colonisation ability of this strain may be ideal as it has been shown to present in the saliva of children with good oral health [28].

Kang et al. [15] reported that the ingestion of *W. cibaria* CMU resulted in 48.2 and 59.4% reduction in VSCs that cause bad breath, hydrogen sulfide, and methyl mercaptan, respectively. In addition, *W. cibaria* CMS1 has been reported to reduce PI by about 20.7% [14]. Additionally, the application of *W. cibaria* CMU to the teeth of beagle dogs for 6 weeks significantly reduced bad breath, PI, and periodontal pathogens [29]. Studies on other *W. cibaria* strains have also reported immunological effects due to their involvement in the production of inflammatory mediators and antimicrobial activity by bacteriocins (e.g., weissellicin) [30].

Suzuki et al. [31] reported that the oil drop containing *Lactobacillus salivarius* WB21 improved the bleeding index among various clinical indicators when tested on 42 subjects for 15 days. In this study, an oral health-related clinical evaluation was carried out after randomised ingestion of *W. cibaria* CMU-containing tablets and placebo for 8 weeks after SRP, and microbial changes in subgingival plaque were evaluated quantitatively. BOP decreased in both groups during the 8 weeks of intervention, and there was a significant difference between the groups in BOP at the maxillary lingual site after 4 weeks and the maxillary buccal site after 8 weeks.

Kumar and Madurantakam [32] reported that the deepness of the pocket is proportional to the probiotic effect. In this study, GI tended to decrease in the probiotic group over time, but there were no significant differences between the groups. In contrast, the difference in PD and PI was not statistically significant between groups. This may be because the subjects were young adults whose pockets are not deep and whose gums are healthy. Iniesta et al. [33] also reported no reduction in GI and PI except for the reduction of *P. gingivialis* after consuming *L. reuteri*-containing tablets for 8 weeks. In another study, *L. reuteri*, an oral probiotic, has been reported to be effective in improving clinical and microbiological parameters in patients with chronic periodontitis when probiotic tablets were administered twice daily for 12 weeks [23]. Therefore, the difference may be due to the experimental design of this study in healthy individuals.

Among several oral bacteria, *F. nucleatum* is known to be involved in the production of VSCs [15]. *F. nucleatum* is present in large numbers in the oral cavity, forms aggregates with other bacteria, and is known to act as a bridge between the primary and secondary settlers on the tooth surface. In the present study, the probiotic group tended to have reduced Gram-negative and Gram-positive bacteria. In particular, a change in the numbers of *F. nucleatum* was statistically significant during the intervention. Change in the numbers of *S. aureus* was statistically significant between the groups during the eight-week intervention. Since *W. cibaria* CMU has been reported to have both a coaggregation ability with *F. nucleatum* and antimicrobial activity against *F. nucleatum* [15], the results of this study demonstrated the antimicrobial activity of *W. cibaria* CMU against *F. nucleatum*.

Oral bacteria are sensitive to various types of oxygen. There are relatively few obligate aerobes in the oral cavity, and most of the earliest colonies on the tooth surface are facultative anaerobes, including *Streptococcus* and *Actinomyces*. As the oral biofilm forms, it quickly becomes anaerobic, and a large proportion of obligate anaerobes colonise the oral cavity. In this study, the probiotic group showed a reduction in the proliferation of the early colony *Streptococcus* group and *A. viscosus* at week 4 without affecting PI.

To the best of our knowledge, our study is the first to evaluate the effects of *W. cibaria* on oral health and microbiota. This study was a well-designed double-blind, placebo-controlled randomised controlled trial evaluating the effects of *W. cibaria* CMU-containing tablets after SRP. In this study, improvement in bleeding index and microbiota were identified in the probiotic group after 8 weeks. The clinical significance of this result was that people with taking *W. cibaria* CMU could improve their BOP index compared to people without taking it. But, in regard to the limitation of this study, participants were healthy people, not patients. Therefore, the results could not be generalized to people with gingival disease. To confirm the extensive clinical effects of *W. cibaria* CMU, further studies should be conducted on patients with gingivitis and other oral diseases. Generally, probiotics are recommended to be used as an adjuvant to mechanical debridement and preventive action, not for sole treatment.

## Conclusions

*W. cibaria* CMU is considered an oral care probiotic that can improve oral health and prevent oral disease. Our study demonstrated that *W. cibaria* treatment could lead to an improvement in the bleeding index and the suppression of propagation of some oral bacteria in people without periodontitis.

## Data Availability

The datasets used and/or analysed during the current study are available from the corresponding author on reasonable request.

## Abbreviations

oraCMU: *Weissella cibaria* CMU
BOP: Bleeding on probing
PD: Probing depth
GI: Gingival index
PI: Plaque index
LAB: Lactic acid bacteria
VSC: Volatile sulfur compound
